# Imaging Mass Cytometry‐Based Immune Profiling of Human Peyer's Patches in Patients with Crohn's Disease

**DOI:** 10.1002/eji.70071

**Published:** 2025-10-06

**Authors:** Adrian Huck, Yasmina Rodriguez‐Sillke, Christian Bojarski, Désirée Kunkel, Anja A. Kühl, Malte Lehmann, Philip Bischoff, Ulrich Steinhoff, Britta Siegmund, Rainer Glauben

**Affiliations:** ^1^ Medical Department Division of Gastroenterology, Infectious Diseases and Rheumatology, Campus Benjamin Franklin Charité – Universitätsmedizin Berlin Corporate Member of Freie Universität Berlin Humboldt‐Universität zu Berlin Berlin Germany; ^2^ Department of Chemistry, Biology, Pharmacy Freie Universität Berlin Berlin Germany; ^3^ Berlin Institute of Health at Charité ‐ Universitätsmedizin Berlin Flow & Mass Cytometry Core Facility Berlin Germany; ^4^ Charité ‐ Universitätsmedizin Berlin, Corporate Member of Freie Universität Berlin, Humboldt‐Universität zu Berlin; iPATH.Berlin, Core Unit of the Charité Berlin Germany; ^5^ Berlin Institute of Health at Charité – Universitätsmedizin Berlin, BIH Academy, Clinician Scientist Program Berlin Germany; ^6^ Charité ‐ Universitätsmedizin Berlin, Institut für Pathologie Berlin Germany; ^7^ Institute For Medical Microbiology and Hospital Hygiene Philipps University of Marburg Marburg Germany

**Keywords:** Crohn's disease, IBD, IMC, lymphoid follicles, mass cytometry, Peyer's patches

## Abstract

Lymphoid follicles in the human gut are critical immune hubs, yet their role in Crohn's disease pathogenesis remains poorly understood. Here, we apply multiplexed imaging mass cytometry to spatially profile Peyer's patches and lymphoid follicles in biopsies from healthy controls and Crohn's disease patients with ileitis, isolated colonic involvement, or in remission. Despite tissue heterogeneity, our optimized preprocessing pipeline enabled robust tissue annotation, single‐cell phenotyping, and neighbourhood‐level analysis. While conventional analysis based on cell frequencies did not distinguish disease states, spatial analysis revealed disease‐associated remodelling of lymphoid architecture. Biopsies from colonic Crohn's disease patients showed, within follicles, increased frequencies of activated CD8⁺ T cells and a reduction in naïve T cells, alongside enrichment of B cell–T cell interaction neighbourhoods. These alterations were most pronounced in smaller B‐cell patches, suggesting more functionally dynamic immune‐cell interactions in compact lymphoid structures. In contrast, Crohn's disease ileitis samples closely resembled healthy tissue, with minimal structural or immune cell perturbations. Our data support a model in which Peyer's patches and lymphoid follicles undergo structural and functional remodelling in response to colonic inflammation. These findings underscore the value of spatially resolved immune profiling to uncover tissue‐specific immune dynamics in inflammatory bowel disease.

## Graphical abstract

Using imaging mass cytometry (IMC), we spatially profiled Peyer's patches and lymphoid follicles in IBD. In colonic Crohn's disease, follicles showed enriched activated CD8⁺ T cells and B–T neighbourhoods—most pronounced in small B‐cell patches—whereas ileitis resembled healthy tissue. Spatial features outperformed simple cell‐frequency readouts.

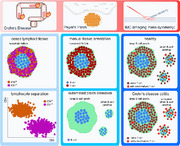

**Abbreviations**
IBD: inflammatory bowel disease
CD: Crohn's disease
UC: ulcerative colitis
PP: Peyer's patches
IMC: imaging mass cytometry
PCA: principal component analysis
LP: lamina propria

## 1 Introduction

Inflammatory bowel disease (IBD) encompasses a group of chronic, relapsing inflammatory disorders of the gastrointestinal tract, primarily comprising Crohn's disease (CD) and ulcerative colitis (UC). While both share certain clinical and pathological features, CD can affect any part of the gastrointestinal tract, though it most commonly involves the terminal ileum and colon, while UC is confined to the colon and rectum. The pathogenesis of IBD is multifactorial, involving a complex interplay between genetic susceptibility, dysregulated immune responses, and environmental factors, leading to chronic inflammation and progressive tissue damage [1].

The ileum, the terminal section of the small intestine, plays a critical role in the immune defence due to its dense microbial exposure and abundant lymphoid tissue. Here, the Peyer's patches (PP) are key immune structures, containing aggregates of lymphoid follicles. These structures are essential for initiating and regulating immune responses to intestinal antigens, including dietary components and commensal microorganisms. PP contribute to mucosal immunity by sampling luminal antigens via specialized epithelial cells known as M cells, and initiate immune responses by activating T and B lymphocytes [2, 3, 4].

In patients with CD, PP in the ileum undergo significant immune dysregulation [5]. Alterations in these lymphoid structures may lead to an exaggerated immune response to intestinal bacteria and contribute to the chronic inflammation characteristic of CD [6]. Further, structural changes in PP may have a direct role in disease pathogenesis. For instance, impaired antigen presentation and dysregulated T‐cell responses in the PP have been implicated in maintaining inflammation in CD [7]. In contrast, UC predominantly affects the colon and typically spares the ileum and its associated lymphoid tissue. PP are increasingly being recognized as potential targets for therapeutic interventions, as modulating their immune responses could help control mucosal inflammation in IBD [8].

Despite their critical role in the pathogenesis of IBD, analysis of PP remains challenging due to their small size, inherent heterogeneity, and complex cellular architecture. These lymphoid aggregates contain specialized M cells, dendritic cells, macrophages, T lymphocytes, and B lymphocytes, which interact dynamically to regulate mucosal immune responses [9, 10]. Their architecture includes distinct zones for antigen sampling and lymphocyte activation [11], forming a highly compartmentalized and dynamic immunological environment. Traditional techniques, limited in multiplexing and spatial resolution, often fail to capture this complexity, making it challenging to fully understand how immune dysregulation within these structures contributes to IBD pathology [12].

Imaging mass cytometry (IMC) enables highly multiplexed, spatially resolved analysis of tissue architecture and immune cell phenotypes. By using metal‐tagged antibodies and mass spectrometry–based detection, IMC allows simultaneous detection of dozens of markers in a single tissue section [13], enabling the analysis of complex lymphoid structures like PP in IBD, where spatial context and cellular diversity are critical for understanding immune dysregulation.

In this study, we applied highly multiplexed IMC to spatially profile PP and isolated lymphoid follicles from intestinal biopsies from CD patients and healthy controls. Using an optimized preprocessing pipeline, we performed single‐cell segmentation, phenotyping, and manual tissue annotation, followed by quantitative analysis of cell frequencies, cell‐cell interactions, and cellular neighbourhood composition. This integrative approach revealed disease‐specific alterations in lymphoid architecture and T cell activation patterns, not detectable by conventional frequency‐based analyses.

## 2 Results

### 2.1 Peyer's Patches from Human Ileal Biopsies: Sampling Constraints and Analytical Strategy

**FIGURE 1:**
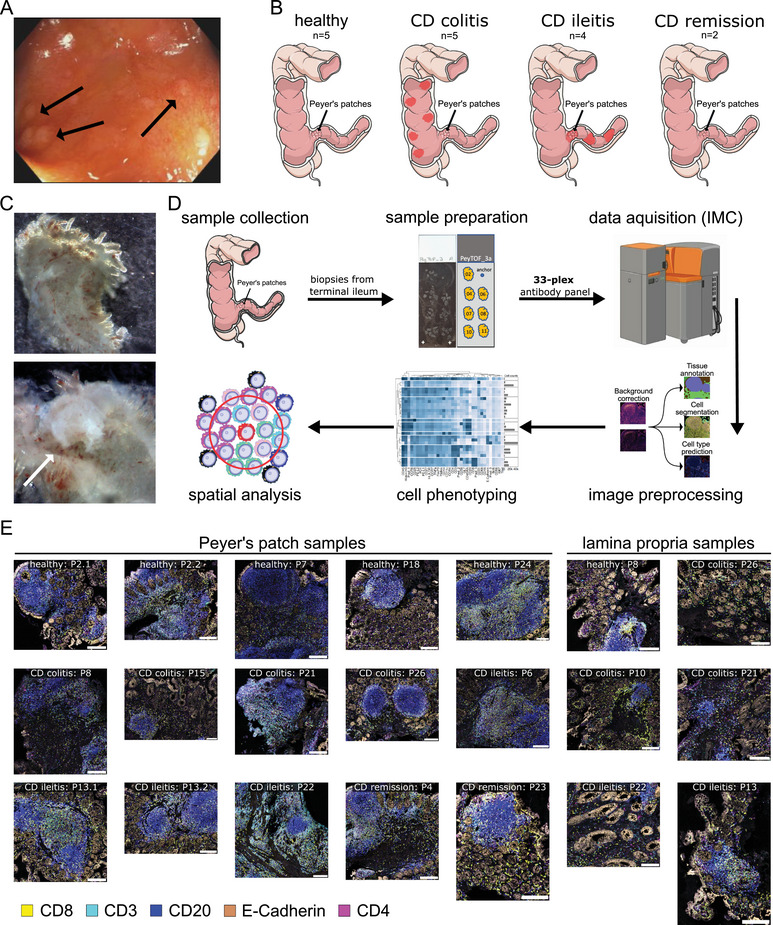
**Spatial profiling of human Peyer's patches reveals heterogeneity in size and tissue composition**. (A) Representative endoscopic image of the terminal ileum highlighting Peyer's patches (arrows). (B) Overview of inflammatory sites among our Crohn's disease (CD) groups, distinguishing CD ileitis (inflammation at the sampling site) from CD colitis (inflammation distal from the sampling site). (C) Stereomicroscope images of biopsy punches without (above) and with (below) follicular structures. (D) Workflow for spatial profiling of formalin‐fixed, paraffin‐embedded ileal biopsies using 33‐plex imaging mass cytometry (IMC), including tissue sectioning, staining, image acquisition, preprocessing, cell phenotyping, and spatial analysis. (E) Annotated lymphoid follicles detected in originally classified Peyer's patch samples (left) and LP‐only samples (right). The figure was in part generated using Servier Medical Art, provided by Servier (Creative Commons Attribution 3.0 Unported) and Milosevic et al. [22] (Creative Commons CC BY).

PP were sampled during ileocolonoscopy from the terminal ileum of healthy controls and CD patients. Patient groups and corresponding tissue samples were stratified based on the anatomical location of inflammation—either distal to the sampling site (CD colitis), exclusively at the sampling site (CD ileitis), or absence of active inflammation at all (CD remission; Table 1; Figure 1A,B). However, macroscopic identification of PP during endoscopy and subsequent microscopic manual preparation proved challenging (Figure 1A,C). Dissociation of biopsies into single‐cell suspension was often limited by the variable lamina propria (LP) content and inconsistent inclusion of follicles. To address these, we first employed a manual dissection protocol for PP isolation [14], which was discontinued due to poor performance on inflamed tissue and limited yields of viable cells, suitable for single‐cell sequencing or high‐dimensional cytometry. Ultimately, we adopted a histology‐guided strategy in which formalin‐fixed, paraffin‐embedded biopsies were first screened for follicular structures using haematoxylin and eosin (H&E) staining, then processed for highly multiplexed IMC to analyze the immune architecture in situ at single‐cell resolution. Our sample processing and analytical workflow included manual arrangement of multiple samples per slide, 33‐marker IMC staining (Table S1, Figure S1) and acquisition, image preprocessing, single‐cell phenotyping, and spatial neighbourhood analysis (Figure 1D). This approach enabled robust characterization of cellular structures across all patient groups despite substantial variability in tissue morphology, especially regarding lymphoid follicle size, shape, or tissue location (Figure 1E).

**TABLE 1:** Overview of patient cohort.

Characteristic	Healthy *N* = 5	CD colitis *N* = 6	CD ileitis *N* = 4	CD remission *N* = 3
Included	2018‐04‐20 to 2019‐06‐26	2018‐07‐24 to 2019‐08‐22	2018‐06‐27 to 2019‐05‐14	2018‐05‐14 to 2019‐05‐22
Include PP	4 (80%)	5 (83%)	3 (75%)	2 (67%)
Location				
* L1‐terminal ileum*	0 (NA%)	0 (0%)	3 (75%)	2 (67%)
* L2‐colon*	0 (NA%)	1 (17%)	0 (0%)	0 (0%)
* L3‐ileocolon*	0 (NA%)	5 (83%)	1 (25%)	1 (33%)

### 2.2 Standardized IMC Analysis of Human Lymphoid Follicles Through Preprocessing and Annotation

**FIGURE 2:**
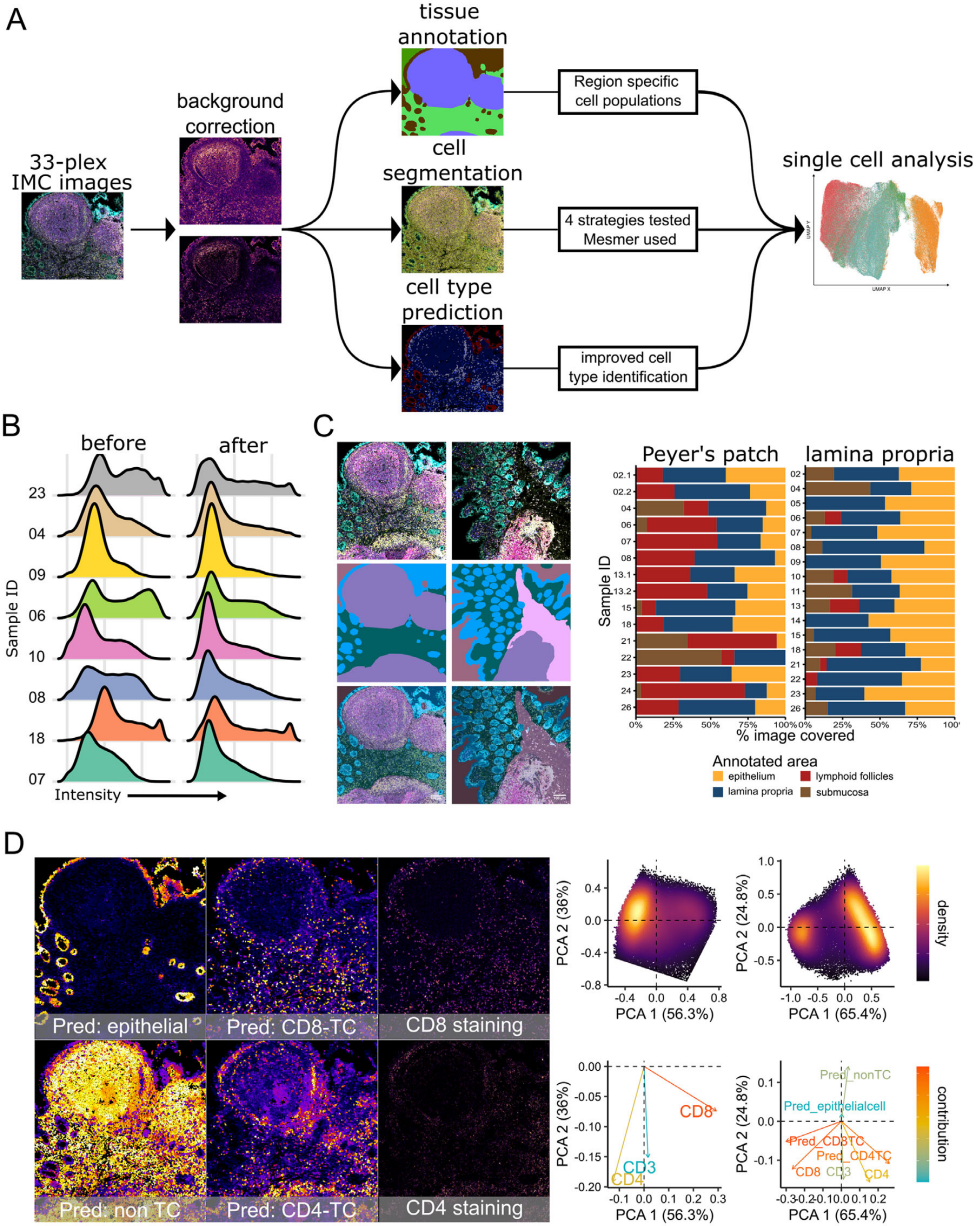
**Optimized preprocessing pipeline enables robust analysis of IMC data from human Peyer's patches**. (A) Schematic overview of the image preprocessing pipeline, including background correction, manual tissue annotation, cell segmentation, and image‐level cell type classification. (B) CD3 intensity distribution across representative samples before (left) and after (right) background correction. (C) Representative multiplexed IMC images (above) used for tissue annotation (Histone H3: white, CD20: red, CD45: blue, CD3: yellow, E‐Cadherin: cyan); centre: annotated tissue compartments; below: overlay of annotation with original image. The right bar graph shows relative abundance of each tissue compartment per sample (excluding background). (D) Pixel‐level class probabilities derived from a random forest classifier trained in ilastik using manual annotations (CD4⁺ T cells, CD8⁺ T cells, non‐T cells, and epithelial cells). These probabilities were added as four new input channels. Right: PCA plots of CD3⁺ cells show cell separation using marker expression only (left) or marker expression plus probability features (right).

To address variability across human biopsy samples, we developed a robust preprocessing pipeline for IMC analysis of human PP, comprising background correction, manual tissue annotation, cell segmentation, and image‐level cell type prediction (Figure 2A). Despite standardized protocols for sample preparation and staining, signal intensity varied markedly between samples. Therefore, the silver mountain operator (SMO) algorithm was applied for background correction [15], improving downstream analysis consistency (Figure 2B). Tissue segment annotation was performed manually using a panel of markers, including Histone H3, CD20, CD45, CD3, and E‐Cadherin, to delineate the LP, lymphoid follicles, epithelium, submucosa, as well as background (Figure 2C).

While conventional cell segmentation methods performed adequately for most tissues, they struggle in lymphoid regions where tightly packed lymphocytes exhibit low cytoplasm‐to‐nucleus ratios. In particular, distinguishing between CD4⁺ and CD8⁺ T cells in lymphoid follicles was challenging. Therefore, a subset of cells was manually annotated by overlapping cell masks onto a subset of the original marker images. Four classes, comprising CD8⁺ T cells, CD4⁺ T cells, non‐T cells, and epithelial cells, were used to train a random forest classifier in ilastik [16], yielding cellmask‐level class probabilities. These probability maps were then added to the original marker channels as additional input layers (Figure 2D, left), significantly enhancing the separation of closely packed lymphocyte subsets (Figure 2D, right).

### 2.3 Cellular Frequencies and Densities Reveal Structural Consistency and Aberrant Mucosal Immune Activation in Crohn's disease

**FIGURE 3:**
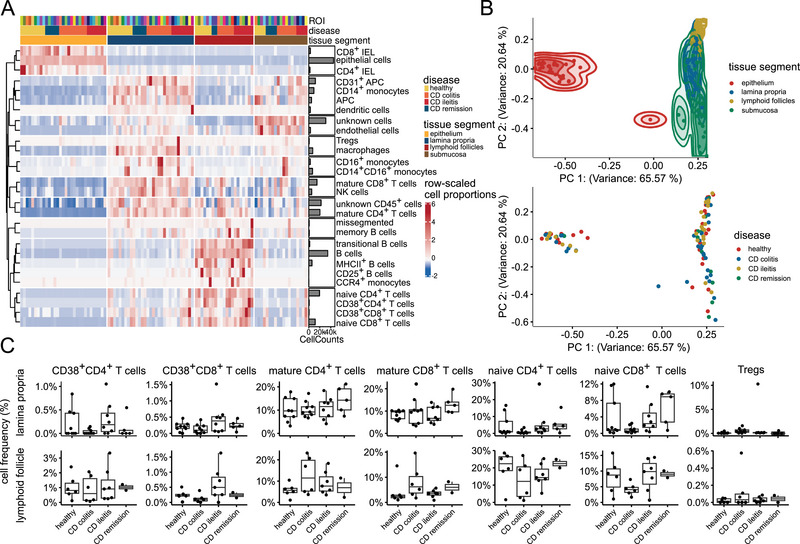
**Human Peyer's patches show distinct cellular composition with increased markers of inflammation in Crohn's disease**. (A) Heatmap showing relative cell frequencies for each sample and annotated tissue segment. (B) Principal component analysis (PCA) of cell frequencies per tissue segment and sample, coloured by tissue segment (top) or disease state (bottom). Samples are either coloured by tissue segment (upper panel) or disease state (lower panel). (C) Frequencies of selected T cell subsets in LP (top) and lymphoid follicles (bottom) for each patient group.

Mean marker expression aligned with expected immune cell signatures (Figure S2A), supporting the reliability of our preprocessing pipeline for single‐cell resolution analysis. Cell frequency analysis across tissue segments, disease states, and patient samples revealed distinct compositional differences between annotated tissue segments, yet did not differentiate between disease states (Figure 3A). Subsequent principal component analysis (PCA) effectively separated tissue segments but failed to provide disease‐specific clustering (Figure 3B).

Cell frequencies and cell densities of major T cell populations demonstrated substantial intra‐group variability (Figure 3C; Figure S2C). Overall, there were no statistically significant differences between patient groups. However, CD colitis displayed the most pronounced shifts for various selected cell types (e.g., CD38^+^CD8^+^ T cells, mature CD8^+^ T cells, naïve CD8^+^ T cells for LP, CD38^+^CD8^+^ T cells, naïve CD4^+^ T cells, naïve CD8^+^ T cells, and Tregs for follicles) as defined in Figure S2B). In contrast, samples from CD ileitis more closely resembled healthy controls than CD colitis, highlighting divergent immunological profiles across disease locations. To further explore tissue architecture and potential disease‐related remodelling, we next performed an analysis of spatial cell‐cell interactions.

### 2.4 Spatial Cell–Cell Interactions Reveal Tissue Compartmentalization and Disease‐Specific Remodelling

**FIGURE 4:**
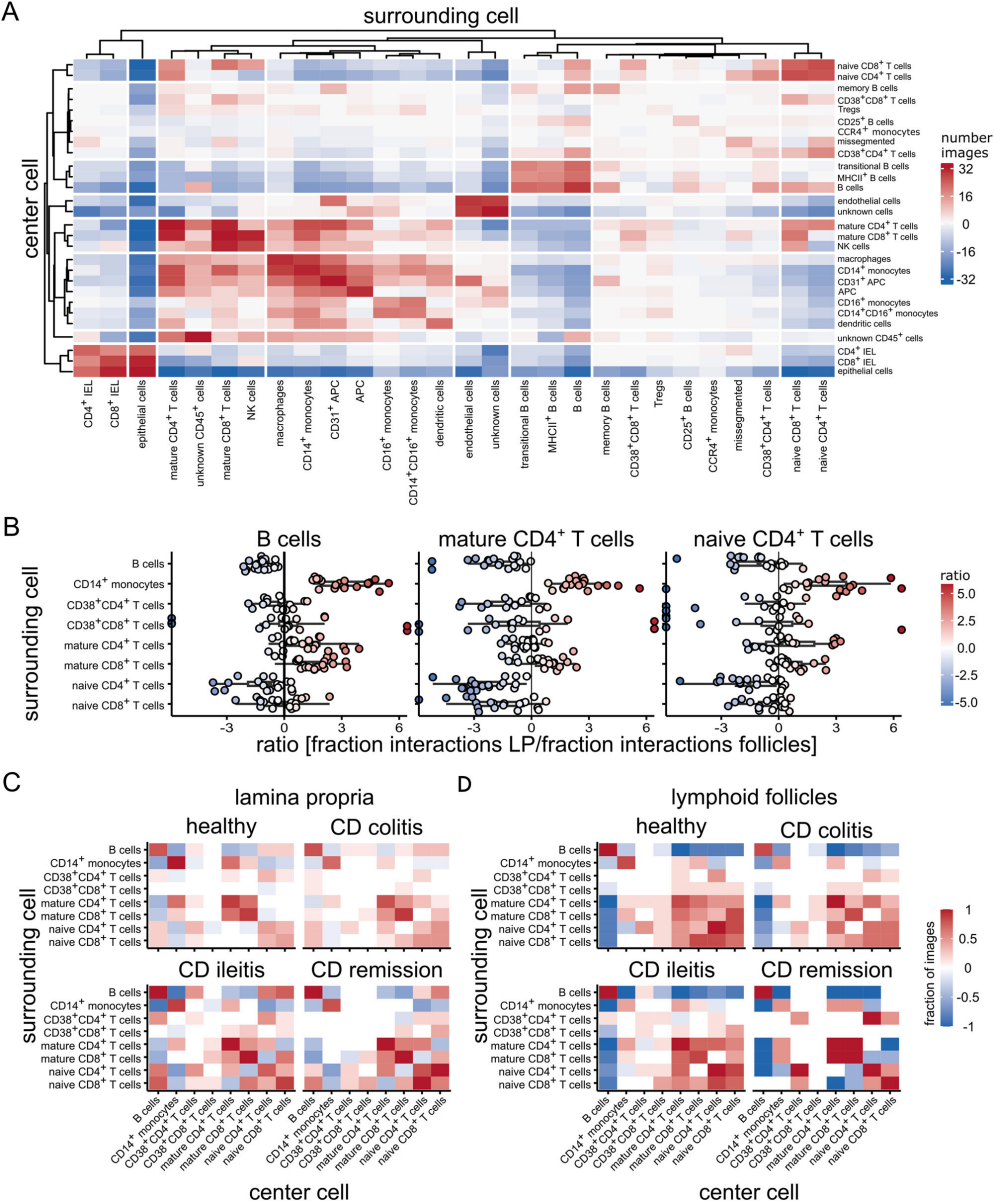
**Cell–cell interaction patterns reveal compartment‐specific organization and disease‐associated alterations**. (A) Global cell–cell interactions calculated across entire images, independent of tissue segmentation. (B) Ratio of interaction frequencies between LP and lymphoid follicles for each cell pair; red indicates enrichment in LP, blue in follicles. (C) Interaction patterns computed within each annotated tissue segment for all patient groups; heatmaps show the number of samples with significant attraction (red) or avoidance (blue) for each cell–cell pair.

Cell–cell interaction patterns were identified by summarizing significant co‐occurrences across images or tissue regions within a maximum distance of 20 µm, as described in the Materials and Methods section. Analysis across all cells, independent of tissue segment annotation (Figure 4A), revealed structured interaction patterns between epithelial cells and intraepithelial CD8⁺ T cells, between naïve T cells and B cells, and between mature CD8⁺ T cells and monocytes, which were spatially consistent across samples. Interactions involving mature CD4⁺ and CD8^+^ T cells, monocytes/macrophages or naïve T cells appeared to localise to specific regions. To assess interaction patterns, we repeated the analysis in LP (Figure S3A) and lymphoid follicle tissues (Figure S3B), and compared interaction frequencies between these regions (Figure 4B). For each interaction, we calculated the ratio of its frequency in LP versus follicles, allowing region‐specific interactions to be visualized as shifts to the left (follicle‐enriched) or right (LP‐enriched) in Figure 4B. Mature CD8⁺ T cell‐monocyte interactions, as well as CD14⁺ monocyte interactions with B cells, naïve T cells, and mature CD4⁺ T cells, showed higher frequencies in the LP. In contrast, interactions enriched in follicles included B cell–naïve T cell and B cell–B cell contacts. These region‐specific patterns were consistent across samples and reflected distinct tissue compartmentalization of cellular interactions.

We next assessed how cell–cell interactions varied by disease state within distinct tissue regions. In the LP (Figure 4C), interaction patterns differed markedly between healthy individuals and patients with CD. CD4⁺ and CD8⁺ T cells in healthy samples showed clear segregation between naïve and mature T cell interactions. In contrast, CD samples exhibited increased cross‐interaction, with more frequent naïve–mature T cell contacts. In the follicular regions (Figure 4D), interaction patterns remained largely consistent across disease states. Cross‐interactions between naïve and mature T cells were already present in healthy individuals and changed only marginally in disease states, indicating minimal disruption of the follicular interaction structure across conditions.

### 2.5 Automated B Cell Patch Detection and Neighbourhood Profiling Uncover Follicle Heterogeneity and Inflammatory State Distinctions

**FIGURE 5:**
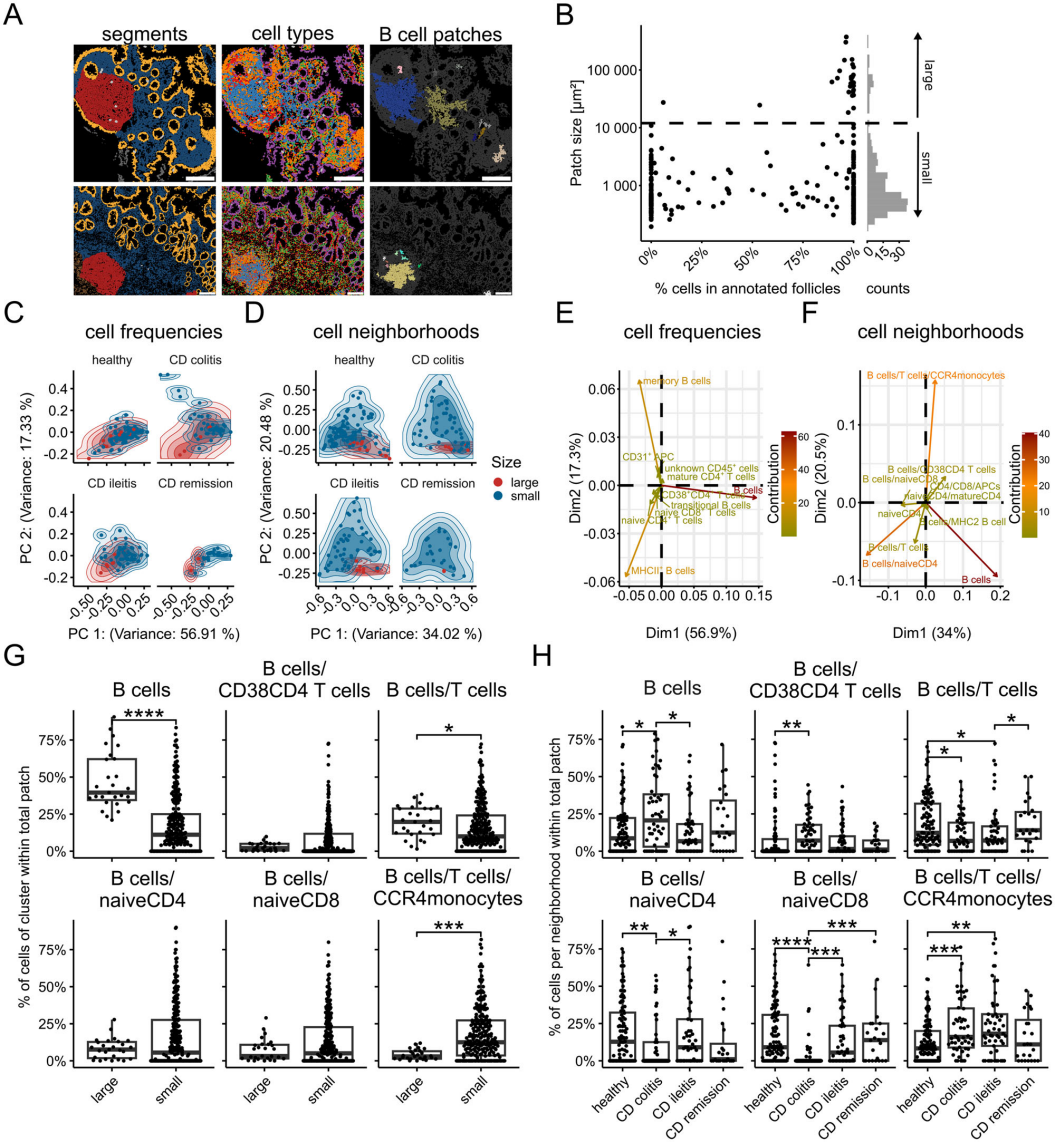
**Cellular neighbourhoods discriminate between small and large B cell patches and reveal disease‐associated alterations in Crohn's colitis**. (A) Representative images from supervised B‐cell patch detection. Left: annotated tissue segments with follicles in red. Middle: major cell populations with B cells in blue. Right: all detected B cell patches displayed in randomly assigned, distinct colours. (B) Tissue localization and size of B cell patches across all disease groups; patches >12,000 µm^2^ were classified as large follicles. (C, D) Plots show principal component analysis (PCA) for each group separately, but are calculated for all disease conditions using either cell type composition (C) or neighbourhood composition (as defined in Figure S4A; Table S2) (D). (E, F) Contribution of features in cell types (E) or neighbourhoods (F), to PCA in (C) and (D), respectively. (G, H) Frequencies of neighbourhood types per patch across follicle sizes (G) or disease groups (H). For group‐wise comparison, the Wilcoxon test with Benjamini–Hochberg correction was used, and only significant results are shown. *p ≤ 0.05, **p ≤ 0.01, ***p ≤ 0.001, ****p ≤ 0.0001.

Given the prominence of B cell centres in PPs, a supervised detection approach to distinguish B cell‐enriched areas from the surrounding T cell zones was applied. Automated B cell patch detection identified discrete B cell patches both within and outside the annotated follicles (Figure 5A; Figure S6). Notably, B cell patches were also detected in samples annotated exclusively as LP, indicating that focal B cell accumulations can occur outside conventionally recognized lymphoid structures. Moreover, while large B cell patches were predominantly located within manually annotated follicular regions, approximately half of the small patches were located outside these areas. This highlights the limited sensitivity of manual annotation for detecting smaller B‐cell‐dense areas and, therefore, potentially missing immunologically relevant lymphoid structures (Figure 5B; Figure S5A).

We compared small B cell patches with larger follicular structures using PCA on cell frequencies and cellular neighbourhood composition within each patch. Cellular neighbourhoods were defined using k‐means clustering of cells according to the fractions of cell clusters in their direct neighbourhood and were annotated based on the enrichment of cell populations (Figure S4A), reflecting known tissue structures (Figure S4B). While cell type frequencies showed minimal separation between small and large B cell regions, PCA using cellular neighbourhoods revealed clear clustering, with large follicles forming a distinct group and small patches displaying greater heterogeneity (Figure 5C,D).

Biplots with feature loadings showed that this separation was driven by T cell‐enriched B cell neighbourhoods (Figure 5E, F). There were significant differences between large and small B cell patches for B cell‐enriched, B cell and T cell‐enriched, as well as T cell and monocyte‐enriched B cell neighbourhoods (Figure 5G).

Focusing the neighbourhood analysis on small B cell patches across disease states, we identified significant shifts in the composition of these neighbourhoods, particularly those enriched for T cells (Figure 5H). In contrast, detected B cell patches did not differ in size between patient groups (Figure S5B). Notably, CD colitis samples showed consistent and significant differences from healthy controls, all the more when we look for Ki‐67 expressing cells in the small B cell patches (Figure S5C). CD ileitis exhibited more subtle changes and distinct patterns from CD colitis, particularly in B cell‐enriched and naïve CD4^+^/mature CD4^+^ T cell‐enriched B cell neighbourhoods.

Our spatially resolved analysis enabled the identification of immune remodelling in PP and LP that was not detectable through cell frequency analysis alone, highlighting the importance of integrating spatial context in uncovering disease‐associated tissue organization in IBD.

## 3 Discussion

We present a robust, spatially resolved analysis pipeline for characterizing immune architecture in human PP and lymphoid follicles, addressing key challenges in sampling and tissue heterogeneity. While conventional readouts, such as marker expression and cell frequency, failed—at least for the given sample number—to distinguish CD subtypes, spatial analysis revealed pronounced differences in T cell activation and lymphoid organization, particularly in colonic CD without ileal involvement (CD colitis). These results highlight the power of high‐dimensional spatial profiling in dissecting immune remodelling in mucosal inflammation.

Robust IMC analysis is complicated by high inter‐sample variability and background signal, which disrupts automated data processing. Variability in tissue preparation, fixation, antigen availability, staining, or instrument calibration can introduce substantial bias, limiting cross‐sample comparability [17]. These issues especially impact automated segmentation and phenotyping algorithms, which rely on consistent signal intensities [18]. To overcome these limitations, we applied background subtraction to eliminate non‐specific signal [19], intensity scaling and transformation to normalize marker distribution [20], and batch correction using internal reference samples [21]. Quality control measures were applied to ensure data integrity across the dataset [22, 23]. These steps enabled accurate single‐cell segmentation, cell type classification, and spatial neighbourhood analysis despite biopsy heterogeneity. By annotating tissue structures and segments, we were able to account for variability in LP abundance, an important confounder that dissociative single‐cell approaches cannot resolve. This annotation also enabled the identification of intra‐ and inter‐segment differences, as recently demonstrated in other tissue settings [24, 25]. Moreover, our preprocessing pipeline, including robust cell type prediction using image‐level marker distribution, enabled reliable analysis of densely cellular lymphoid regions, which are challenging in high‐dimensional imaging. To address spatial spillover, a known limitation of multiplexed antibody‐based imaging resulting in artefactual co‐expression of mutually exclusive markers, recent solutions like REDSEA [26] and deep‐learning‐based classifiers [27] have been proposed. While we applied a more focused strategy tailored to our tissue and panel (e.g., the discrimination of CD8^+^ and CD4^+^ T cells in densely packed lymphoid follicles), these advances highlight the growing need for context‐aware segmentation and classification methods in spatial proteomics.

Applying cell‐cell interaction analysis, we could demonstrate clear separations of certain cell types, e.g., epithelial cells predominantly interact with epithelial cells. Immunologically, this approach revealed distinct avoidance patterns in the lamina propria of healthy patients, particularly between naïve and mature T cells. In contrast, in Crohn's disease patients, these cells gain attraction: the clear separation between immunological structures is lost, and interactions between mature and naïve T cells (e.g., via cytokine‐mediated effects) become highly probable. Comparable findings were reported by Kondo et al. [28] for regulatory and activated (CD38⁺) T cells, further supporting the relevance of such interaction analyses in uncovering otherwise hidden immunological alterations. Spatial neighbourhood analysis has proven valuable across diverse biological contexts for revealing tissue organization and function. In tumour biology, it has delineated fibroblast subtypes and immune cell neighbourhoods shaping local immune responses and disease progression [29, 30]. Applying this concept to intestinal inflammation, we found that neighbourhood‐level features in large lymphoid follicles revealed differences in cellular activity that were not captured by frequency‐based analyses alone.

We noted higher frequencies of activated CD38^+^CD8^+^ T cells and lower frequencies of naïve CD4^+^ and CD8^+^ T cells in both LP and PP of CD colitis samples compared with healthy controls and CD ileitis. In contrast, CD ileitis appeared more similar to the healthy state, with relatively higher frequencies of naïve T cells and fewer activated CD38^+^CD8^+^ T cells. These trends suggest that, despite the absence of ileal inflammation in CD colitis, immune alterations may still be present at the site of the PP.

In line with these observations, Visekruna et al. [31] reported that in CD, T cells within PP fail to undergo apoptosis due to upregulation of survival pathways (e.g., Bcl‐2), resulting in the accumulation of hyperactivated, diet‐reactive CD4^+^ T cells.

This concept is supported by Rodríguez‐Sillke et al. [32], who demonstrated that small intestinal inflammation is associated with the presence of systemic food antigen‐specific CD4^+^ T cells exhibiting enhanced cytokine responses. This aligns with our observation that CD colitis patients display elevated frequencies of activated CD38^+^ T cells and reduced naïve T cells at the site of the PP, suggesting a systemic or pre‐activated immune state.

Interestingly, our spatial analysis revealed that smaller B cell patches exhibited greater variability in neighbourhood composition across disease states compared with larger PP, suggesting that these structures support more dynamic immune cell interactions and potentially stronger local immune responses, further indicated by increased Ki‐67+ cell frequency as a marker for cell proliferation. This aligns with previous studies highlighting the active role of isolated lymphoid follicles in mucosal immunity [33], although in inflamed tissue, some aggregates may also represent tertiary lymphoid structures.

Spatial neighbourhood analysis further revealed structural remodelling of small lymphoid follicles in CD colitis, as shown in other studies. These analyses further underscore the structural and cellular heterogeneity of intestinal lymphoid follicles, identifying distinct inner and outer follicle neighbourhoods with different B and CD4⁺ T cell levels based on follicle maturity [34] and describing activated T and B cell populations within germinal centre‐like structures in ulcerative colitis [35].

Our study revealed that CD colitis samples showed increased frequencies of B cell‐enriched and activated CD4^+^ T cell‐enriched neighbourhoods and reduced neighbourhoods dominated by naïve CD4^+^ or CD8^+^ T cells. These spatial shifts indicate a reorganization of immune architecture within follicles, favouring interactions between effector T cell–B cells. Fujimura et al. [36] also reported greater immunological activity in large dome‐type follicles due to their association with M cells and follicle‐associated epithelium. In contrast, Rubio et al. [37] reported increased lymphoid aggregates and plasma cells in the LP of CD, but fewer activated lymphocytes within this tissue compared with healthy tissue.

Furthermore, spatial transcriptomic analysis by Cohen et al. revealed that even small isolated lymphoid follicles in mouse models exhibit enriched immune activity, highlighting the functional compartmentalization and potential immunological relevance of small follicles in the gut mucosa [38]. This may reflect a redistribution of effector cells away from organized lymphoid structures into the surrounding tissue, driven by systemic inflammatory cues.

Recent landmark single‐cell studies have revealed the cellular complexity and inflammatory remodelling of the intestinal mucosa in IBD [39, 40, 41, 42]. However, these dissociative approaches inherently lack spatial resolution and do not capture how immune populations are organized within the tissue microenvironment. While spatial transcriptomics is a rapidly advancing technology, recent studies regarding mucosal immunity [43, 44, 45] have not yet addressed human IBD in depth, nor investigated T cell activation states or structural differences between small and large lymphoid follicles. High‐dimensional imaging approaches, including IMC, are beginning to map spatial immune architectures in the gut, though the follicular microenvironment remains insufficiently explored [28, 34].

Our data highlight that lymphoid follicles represent structurally and functionally distinct compartments, and suggest that both follicle size and location may influence local immune activity in Crohn's disease. These findings underscore the importance of spatial context for interpreting mucosal immune responses and advocate for more integrated approaches that combine single‐cell and spatial analyses to better understand the architecture of intestinal inflammation.

### 3.1 Data Limitations and Perspectives

This study is limited by the small number of patient samples per group, largely due to the difficulty of reliably obtaining PP tissue from inflamed ileal areas. In CD ileitis, local inflammation often obscures PP, reducing the success rate of targeted biopsy collection. Consequently, our analysis lacks a systematic comparison between inflamed and non‐inflamed PP within patients. In addition, our cohort was not treatment‐naïve, and diverse prior or ongoing therapies may have influenced mucosal immune profiles. While our findings reveal robust spatial differences between disease states, these insights are exploratory and not powered for definitive statistical conclusions. To address this, future studies will require more standardized sampling protocols and larger cohorts. Importantly, our data can now inform formal sample size estimation for spatial analysis, as outlined by Bost et al. [46], enabling more targeted experimental design in follow‐up studies. We anticipate that combining spatial profiling with improved recovery of PP under inflammatory conditions will be critical for dissecting the role of local versus systemic immune remodelling in IBD.

## 4 Materials and Methods

### 4.1 Patients and Controls

Adult Crohn's disease (CD) patients and healthy controls were recruited at the Medical Department for Gastroenterology, Infectious Diseases, and Rheumatology, Charité ‐ Universitätsmedizin Berlin. Inclusion criteria for patients were a confirmed diagnosis of CD, either in an active flare or remission. Healthy controls were individuals undergoing routine endoscopy. After informed consent, terminal ileum biopsies were taken, and PP were identified and extracted where possible.

### 4.2 Histopathology

Biopsies were transferred to 10% formalin, fixed overnight at room temperature, and embedded in paraffin. Sections were freshly cut for histochemistry (1–2 µm) or IMC (4 µm). For histological evaluation, sections were dewaxed, H&E‐stained, and mounted with Histokitt (Roth, Karlsruhe, Germany).

### 4.3 Imaging Mass Cytometry

Dewaxed sections underwent antigen retrieval (pH 9.0), blocking, and overnight staining with metal‐conjugated antibodies (Table S1) either purchased pre‐conjugated to metal isotopes (Standard BioTools, South San Francisco, CA, USA) or conjugated in‐house using the MaxPar X8 kit (Standard BioTools). Nuclei were stained with Intercalator‐Iridium (Standard BioTools), slides were rinsed, air‐dried, and stored until acquisition. Samples were analyzed on a Hyperion Imaging System (Standard BioTools). After tuning, regions of interest were selected on panorama images, and laser ablation was performed at 1 µm resolution. Signal spillover was corrected using the CATALYST R package (v1.14.0) [47].

### 4.4 Preprocessing Pipeline

#### 4.4.1 Background Correction

Background correction was performed on single‐channel images using the SMO [15] in a custom Python script (v3.9) with the following settings: sigma: 1.3, size: 7, shape: (1024, 1024), and threshold: 0.1.

#### 4.4.2 Tissue and Cell Segmentation

Tissue segments (LP, lymphoid follicles, etc.) were manually annotated in GIMP (v2.10.24), based on 5‐channel overlays for CD20, E‐Cadherin, CD3, DNA, and CD45. Masks were exported and converted in Fiji (2.16.0/1.54p) for downstream use. The size of each annotated segment was calculated using the “measure regionprops” function from the Steinbock toolkit [23]. Cell segmentation was performed using the MESMER model (1 µm/pixel) at DeepCell.org [48] with merged nuclear (DNA1, Histone H3) and cytoplasmic markers (CD45, E‐Cadherin) (Cellprofiler 4.2.1) [49].

#### 4.4.3 T Cell Prediction

To improve T cell identification, centred image crops (x: 150–450px; y: 150–450px) containing key markers (CD3, CD4, CD8, CD45, CD20, E‐Cadherin, DNA, Histone H3 as well as merged CD45/E‐Cadherin) were exported via the Spectre package (v1.0.0) [50]. Crops and cell masks were annotated in ilastik (v1.3.3post2, Heidelberg, Germany) [16] into CD4⁺, CD8⁺, non‐T, and epithelial cells. A random forest classifier was trained and applied batch‐wise to whole‐slide images. Resulting probability maps were added as additional channels and integrated into the single‐cell dataset for downstream analysis.

#### 4.4.4 Single‐Cell Analysis and Phenotyping

Whole‐cell segmentation masks and marker images were loaded into Spectre to calculate mean marker expression per cell. Cells were assigned to tissue regions based on xy‐position and tissue segmentation masks. Intensities were asinh‐normalized (cofactor 1) and clipped to the first–99th percentile, and cells were filtered by size (15–250 µm^2^). Data were converted into a spatial experiment object for use in the imcRtools (v1.8.0) workflow [23]. Cells were manually pregated using CD45 and E‐Cadherin into major populations and further classified using CD3 and CD20. Within gates, unsupervised clusters were defined using population‐specific markers and merged based on similarity. Cell types were annotated and visualized using UMAP (Uniform Manifold Approximation and Projection).

### 4.5 Spatial Analysis

#### 4.5.1 Interaction Analysis

Cell‐cell interactions were identified by comparing the observed number of cell co‐occurrences within 20 µm against the distribution of co‐occurrences by random shuffling of the cell‐labels as described in the histocat‐package [18] and implemented in the end‐to‐end workflow for multiplexed image processing and analysis [23]. Cell–cell interactions were computed from Delaunay triangulation (≤20 µm) using imcRtools. Interaction analyses were performed globally and within annotated tissue regions (e.g., LP vs. follicles). Attraction and avoidance patterns were summarized, and log_2_‐ratios of interaction partners were used to assess regional preferences.

#### 4.5.2 Cellular Neighbourhoods

Neighbourhoods were defined using a 20 µm interaction radius. A cluster sweep was performed to determine the optimal number of clusters, followed by k‐means clustering (40 centres) on neighbourhood compositions. Resulting clusters were annotated in two levels: first by dominant immune cell populations (e.g., B cell‐rich, T cell‐rich), and second by additional co‐enriched cell types (e.g., B cell‐rich with activated T cells, Table S2).

#### 4.5.3 Patch‐Detection

B cell patches were defined as clusters of ≥10 interacting B cells, identified using the Delaunay‐based interaction graph from the cell–cell analysis and expanded by 1 pixel (≘ 1 µm). All B cell subpopulations were pooled for patch detection. Patch area was calculated, and those >12,000 µm^2^ were classified as large. For each patch, the frequencies of constituent cell types and neighbourhoods were computed.

#### 4.5.4 Statistical Tests/Analysis

Analyses were conducted in R (v4.4.2) using tidyverse (v2.0.0) and ggpubr (v0.6.0). Wilcoxon tests with Benjamini–Hochberg correction were used for group comparisons, and a boxplot generated using ggplot2 (3.5.2) shows the median, the interquartile range (IQR), and 1.5*IQR. PCA and feature contributions were calculated using the stats and factoextra (v1.0.7) packages. Heatmaps were generated using ComplexHeatmap (v2.22.0) [51].

## Author Contributions

Adrian Huck developed and performed data analysis. Yasmina Rodriguez‐Sillke designed and conducted the experiments. Christian Bojarski acquired samples. Désirée Kunkel, Anja A. Kühl, and Malte Lehmann contributed to assay development and analytical validation. Philip Bischoff assisted with tissue annotation. Ulrich Steinhoff, Britta Siegmund, and Rainer Glauben planned and designed experiments, interpreted data, and wrote the manuscript.

## Conflicts of Interest

The authors declare no conflicts of interest.

## Peer Review

The peer review history for this article is available at https://publons.com/publon/10.1002/eji.70071.

## Ethics Statement

All procedures involving human participants were conducted in accordance with the Declaration of Helsinki and approved by the Charité ‐ Universitätsmedizin Berlin Ethics Committee (approval number: EA4/097/14).

## Patient Consent Statement

Written informed consent was obtained from all participants prior to inclusion in the study.

## Supporting information




**Supporting File 1**: eji70071‐sup‐0001‐SuppMat.pdf

## Data Availability

All data supporting the findings of this study are available at Zenodo: https://doi.org/10.5281/zenodo.17159650.

## References

[1] R. J. Xavier and D. K. Podolsky , “Unravelling the Pathogenesis of Inflammatory Bowel Disease,” Nature 448, no. 7152 (2007): 427–434. 17653185 10.1038/nature06005

[2] P. Brandtzaeg and K. Bjerke , “Immunomorphological Characteristics of human Peyer's Patches,” Digestion 46, no. Suppl2 (1990): 262–273. 2124558 10.1159/000200396

[3] K. Fujihashi , T. Dohi , P. D. Rennert , et al., “Peyer's Patches Are Required for Oral Tolerance to Proteins,” Proceedings of the National Academy of Sciences 98, no. 6 (2001): 3310–3315. 10.1073/pnas.061412598 PMC30650 11248075

[4] R. L. Owen and A. L. Jones , “Epithelial Cell Specialization Within Human Peyer's Patches: An Ultrastructural Study of Intestinal Lymphoid Follicles,” Gastroenterology 66, no. 2 (1974): 189–203. 4810912

[5] S. Y. Salim , M. A. Silva , A. V. Keita , et al., “CD83+CCR7‐ dendritic Cells Accumulate in the Subepithelial Dome and Internalize Translocated Escherichia coli HB101 in the Peyer's Patches of Ileal Crohn's Disease,” American Journal of Pathology 174, no. 1 (2009): 82–90. 19095953 10.2353/ajpath.2009.080273 PMC2631321

[6] R. B. Sartor , “Mechanisms of Disease: Pathogenesis of Crohn's Disease and Ulcerative Colitis,” National Clinical Practice Gastroenterology Hepatology 3, no. 7 (2006): 390–407. 10.1038/ncpgasthep0528 16819502

[7] E. Gullberg and J. D. Soderholm , “Peyer's Patches and M Cells as Potential Sites of the Inflammatory Onset in Crohn's Disease,” Annals of New York Academy of Sciences 1072 (2006): 218–232. 10.1196/annals.1326.028 17057202

[8] C. Jung , J. P. Hugot , and F. Barreau , “Peyer's Patches: The Immune Sensors of the Intestine,” International Journal of Inflammation 2010, no. 1 (2010): 823710. 21188221 10.4061/2010/823710 PMC3004000

[9] A. M. Mowat , “Anatomical Basis of Tolerance and Immunity to Intestinal Antigens,” Nature Reviews Immunology 3, no. 4 (2003): 331–341. 10.1038/nri1057 12669023

[10] A. Reboldi and J. G. Cyster , “Peyer's Patches: Organizing B‐cell Responses at the Intestinal Frontier,” Immunological Reviews 271, no. 1 (2016): 230–245. 27088918 10.1111/imr.12400 PMC4835804

[11] H. Lelouard , M. Fallet , B. de Bovis , S. Meresse , and J. P. Gorvel , “Peyer's Patch Dendritic Cells Sample Antigens by Extending Dendrites Through M Cell‐Specific Transcellular Pores,” Gastroenterology 142, no. 3 (2012): 592–601. 22155637 10.1053/j.gastro.2011.11.039

[12] J. I. Park , S. W. Cho , J. H. Kang , and T. E. Park , “Intestinal Peyer's Patches: Structure, Function, and in Vitro Modeling,” Journal of Tissue Engineering and Regenerative Medicine 20, no. 3 (2023): 341–353. 10.1007/s13770-023-00543-y PMC10117255 37079198

[13] C. Giesen , H. A. Wang , D. Schapiro , et al., “Highly Multiplexed Imaging of Tumor Tissues With Subcellular Resolution by Mass Cytometry,” Nature Methods 11, no. 4 (2014): 417–422. 24584193 10.1038/nmeth.2869

[14] P. B. Jorgensen , T. M. Fenton , U. M. Morbe , et al., “Identification, Isolation and Analysis of human Gut‐Associated Lymphoid Tissues,” Nature Protocols 16, no. 4 (2021): 2051–2067. 33619391 10.1038/s41596-020-00482-1

[15] M. Silberberg and H. E. Grecco , “Robust and Unbiased Estimation of the Background Distribution for Automated Quantitative Imaging,” Journal of the Optical Society of America. A, Optics, image science, and vision 40, no. 4 (2023): C8–C15. 10.1364/JOSAA.477468 37132946

[16] S. Berg , D. Kutra , T. Kroeger , et al., “ilastik: Interactive Machine Learning for (bio)Image Analysis,” Nature Methods 16, no. 12 (2019): 1226–1232. 31570887 10.1038/s41592-019-0582-9

[17] S. C. Bendall , E. F. Simonds , P. Qiu , et al., “Single‐cell Mass Cytometry of Differential Immune and Drug Responses Across a human Hematopoietic Continuum,” Science 332, no. 6030 (2011): 687–696. 21551058 10.1126/science.1198704 PMC3273988

[18] D. Schapiro , H. W. Jackson , S. Raghuraman , et al., “histoCAT: Analysis of Cell Phenotypes and Interactions in Multiplex Image Cytometry Data,” Nature Methods 14, no. 9 (2017): 873–876. 28783155 10.1038/nmeth.4391 PMC5617107

[19] M. E. Ijsselsteijn , A. Somarakis , B. P. F. Lelieveldt , T. Hollt , and N. de Miranda , “Semi‐Automated Background Removal Limits Data Loss and Normalizes Imaging Mass Cytometry Data,” Cytometry Part A: the journal of the International Society for Analytical Cytology 99, no. 12 (2021): 1187–1197. 34196108 10.1002/cyto.a.24480 PMC9542015

[20] S. Van Gassen , B. Gaudilliere , M. S. Angst , Y. Saeys , and N. Aghaeepour , “CytoNorm: A Normalization Algorithm for Cytometry Data,” Cytometry Part A: the journal of the International Society for Analytical Cytology 97, no. 3 (2020): 268–278. 31633883 10.1002/cyto.a.23904 PMC7078957

[21] R. Casanova , S. Xu , P. Bost , et al., “Standardization of Suspension and Imaging Mass Cytometry Single‐Cell Readouts for Clinical Decision Making,” Cytometry Part A: the journal of the International Society for Analytical Cytology 107, no. 6 (2025): 390–403. 40407218 10.1002/cyto.a.24940

[22] V. Milosevic , “Different Approaches to Imaging Mass Cytometry Data Analysis,” Bioinformatics Advances 3, no. 1 (2023): vbad046. 37092034 10.1093/bioadv/vbad046 PMC10115470

[23] J. Windhager , V. R. T. Zanotelli , D. Schulz , et al., “An End‐to‐end Workflow for Multiplexed Image Processing and Analysis,” Nature Protocols 18, no. 11 (2023): 3565–3613. 37816904 10.1038/s41596-023-00881-0

[24] S. E. Korman , G. Vissers , M. A. J. Gorris , et al., “Artificial Intelligence‐Based Tissue Segmentation and Cell Identification in Multiplex‐stained Histological Endometriosis Sections,” Human Reproduction 40, no. 3 (2025): 450–460. 39724530 10.1093/humrep/deae267 PMC11879173

[25] R. S. Sandler , J. J. Hansen , A. F. Peery , J. T. Woosley , J. A. Galanko , and T. O. Keku , “Intraepithelial and Lamina Propria Lymphocytes Do Not Correlate with Symptoms or Exposures in Microscopic Colitis,” Clinical and Translational Gastroenterology 13, no. 3 (2022): e00467. 35166714 10.14309/ctg.0000000000000467 PMC8963857

[26] Y. Bai , B. Zhu , X. Rovira‐Clave , et al., “Adjacent Cell Marker Lateral Spillover Compensation and Reinforcement for Multiplexed Images,” Frontiers in Immunology 12 (2021): 652631. 34295327 10.3389/fimmu.2021.652631 PMC8289709

[27] J. L. Rumberger , N. F. Greenwald , J. S. Ranek , et al., “Automated Classification of Cellular Expression in Multiplexed Imaging Data With Nimbus,” BioRxiv (2024). 10.1038/s41592-025-02826-9 PMC13317004 41062826

[28] A. Kondo , S. Ma , M. Y. Y. Lee , et al., “Highly Multiplexed Image Analysis of Intestinal Tissue Sections in Patients with Inflammatory Bowel Disease,” Gastroenterology 161, no. 6 (2021): 1940–1952. 34529988 10.1053/j.gastro.2021.08.055 PMC8606000

[29] Y. Liu , A. Sinjab , J. Min , G. Han , F. Paradiso , and Y. Zhang , “Conserved Spatial Subtypes and Cellular Neighborhoods of Cancer‐associated Fibroblasts Revealed by Single‐cell Spatial Multi‐Omics,” Cancer Cell 43, no. 5 (2025): 905–24 e6. 10.1016/j.ccell.2025.03.004 PMC12074878 40154487

[30] C. M. Schurch , S. S. Bhate , G. L. Barlow , et al., “Coordinated Cellular Neighborhoods Orchestrate Antitumoral Immunity at the Colorectal Cancer Invasive Front,” Cell 183, no. 3 (2020): 838. 33125896 10.1016/j.cell.2020.10.021 PMC7658307

[31] A. Visekruna , S. Hartmann , Y. R. Sillke , et al., “Intestinal Development and Homeostasis Require Activation and Apoptosis of Diet‐Reactive T Cells,” Journal of Clinical Investigation 129, no. 5 (2019): 1972–1983. 30939122 10.1172/JCI98929 PMC6486345

[32] Y. Rodriguez‐Sillke , M. Schumann , D. Lissner , et al., “Analysis of Circulating Food Antigen‐Specific T‐Cells in Celiac Disease and Inflammatory Bowel Disease,” International Journal of Molecular Sciences 24, no. 9 (2023): 8153. 37175860 10.3390/ijms24098153 PMC10179603

[33] B. R. Glaysher and N. A. Mabbott , “Isolated Lymphoid Follicle Maturation Induces the Development of Follicular Dendritic Cells,” Immunology 120, no. 3 (2007): 336–344. 17163957 10.1111/j.1365-2567.2006.02508.x PMC2265896

[34] J. W. Hickey , W. R. Becker , S. A. Nevins , et al., “Organization of the Human Intestine at Single‐Cell Resolution,” Nature 619, no. 7970 (2023): 572–584. 37468586 10.1038/s41586-023-05915-x PMC10356619

[35] D. R. Holman , S. J. S. Rubin , M. Ferenc , et al., “Automated Spatial Omics Landscape Analysis Approach Reveals Novel Tissue Architectures in Ulcerative Colitis,” Scientific Reports 14, no. 1 (2024): 18934. 39147769 10.1038/s41598-024-68397-5 PMC11327370

[36] Y. Fujimura , M. Hosobe , and T. Kihara , “Ultrastructural Study of M Cells From Colonic Lymphoid Nodules Obtained by Colonoscopic Biopsy,” Digestive Diseases and Sciences 37, no. 7 (1992): 1089–1098. 1618058 10.1007/BF01300292

[37] C. A. Rubio , J. Asmundsson , P. Silva , C. Illies , J. Hartman , and L. Kis , “Lymphoid Aggregates in Crohn's Colitis and Mucosal Immunity,” Virchows Archiv 463, no. 5 (2013): 637–642. 23979405 10.1007/s00428-013-1474-5

[38] N. Cohen , H. Massalha , S. Ben‐Moshe , et al., “Spatial Gene Expression Maps of the Intestinal Lymphoid Follicle and Associated Epithelium Identify Zonated Expression Programs,” Plos Biology 19, no. 10 (2021): e3001214. 34634036 10.1371/journal.pbio.3001214 PMC8530339

[39] J. Kinchen , H. H. Chen , K. Parikh , et al., “Structural Remodeling of the Human Colonic Mesenchyme in Inflammatory Bowel Disease,” Cell 175, no. 2 (2018): 372–86 e17. 10.1016/j.cell.2018.08.067 PMC6176871 30270042

[40] L. Kong , V. Pokatayev , A. Lefkovith , et al., “The Landscape of Immune Dysregulation in Crohn's disease Revealed Through Single‐cell Transcriptomic Profiling in the Ileum and Colon,” Immunity 56, no. 2 (2023): 444–58 e5. 10.1016/j.immuni.2023.01.002 PMC9957882 36720220

[41] J. C. Martin , C. Chang , G. Boschetti , et al., “Single‐Cell Analysis of Crohn's Disease Lesions Identifies a Pathogenic Cellular Module Associated With Resistance to Anti‐TNF Therapy,” Cell 178, no. 6 (2019): 1493–508.e20. 10.1016/j.cell.2019.08.008 PMC7060942 31474370

[42] C. S. Smillie , M. Biton , J. Ordovas‐Montanes , et al., “Intra‐ and Inter‐Cellular Rewiring of the Human Colon During Ulcerative Colitis,” Cell 178, no. 3 (2019): 714–30 e22. 10.1016/j.cell.2019.06.029 PMC6662628 31348891

[43] A. Garrido‐Trigo , A. M. Corraliza , M. Veny , et al., “Macrophage and Neutrophil Heterogeneity at Single‐Cell Spatial Resolution in human Inflammatory Bowel Disease,” Nature Communications 14, no. 1 (2023): 4506. 10.1038/s41467-023-40156-6 PMC10372067 37495570

[44] S. M. Parigi , L. Larsson , S. Das , et al., “The Spatial Transcriptomic Landscape of the Healing Mouse Intestine Following Damage,” Nature Communications 13, no. 1 (2022): 828. 10.1038/s41467-022-28497-0 PMC8837647 35149721

[45] E. Mennillo , Y. J. Kim , G. Lee , et al., “Single‐cell and Spatial Multi‐Omics Highlight Effects of Anti‐integrin Therapy Across Cellular Compartments in Ulcerative Colitis,” Nature Communications 15, no. 1 (2024): 1493. 10.1038/s41467-024-45665-6 PMC10876948 38374043

[46] P. Bost , D. Schulz , S. Engler , C. Wasserfall , and B. Bodenmiller , “Optimizing Multiplexed Imaging Experimental Design Through Tissue Spatial Segregation Estimation,” Nature Methods 20, no. 3 (2023): 418–423. 36585456 10.1038/s41592-022-01692-z PMC9998266

[47] S. Chevrier , H. L. Crowell , V. R. T. Zanotelli , S. Engler , M. D. Robinson , and B. Bodenmiller , “Compensation of Signal Spillover in Suspension and Imaging Mass Cytometry,” Cell Systems 6, no. 5 (2018): 612–20 e5. 10.1016/j.cels.2018.02.010 PMC5981006 29605184

[48] N. F. Greenwald , G. Miller , E. Moen , et al., “Whole‐cell Segmentation of Tissue Images With Human‐Level Performance Using Large‐Scale Data Annotation and Deep Learning,” Nature Biotechnology 40, no. 4 (2022): 555–565. 10.1038/s41587-021-01094-0 PMC9010346 34795433

[49] D. R. Stirling , M. J. Swain‐Bowden , A. M. Lucas , A. E. Carpenter , B. A. Cimini , and A. Goodman , “CellProfiler 4: Improvements in Speed, Utility and Usability,” BMC Bioinformatics 22, no. 1 (2021): 433. 34507520 10.1186/s12859-021-04344-9 PMC8431850

[50] T. M. Ashhurst , F. Marsh‐Wakefield , G. H. Putri et al., “Integration, Exploration, and Analysis of High‐Dimensional Single‐Cell Cytometry Data Using Spectre,” Cytometry Part A: The Journal of the International Society for Analytical Cytology 101, no. 3 (2022): 237–253. 33840138 10.1002/cyto.a.24350

[51] Z. Gu , R. Eils , and M. Schlesner , “Complex Heatmaps Reveal Patterns and Correlations in Multidimensional Genomic Data,” Bioinformatics 32, no. 18 (2016): 2847–2849. 27207943 10.1093/bioinformatics/btw313

