# Supplementary material for: Imaging Mass Cytometry‐Based Immune Profiling of Human Peyer's Patches in Patients with Crohn's Disease
Source: Eur J Immunol. 2025 Oct 6;55(10):e70071. doi: 10.1002/eji.70071 (PMC12501404; doi:10.1002/eji.70071)
Supplement: Supplementary file 1 — Supporting File 1: eji70071‐sup‐0001‐SuppMat.pdf [file EJI-55-e70071-s001.pdf]

A

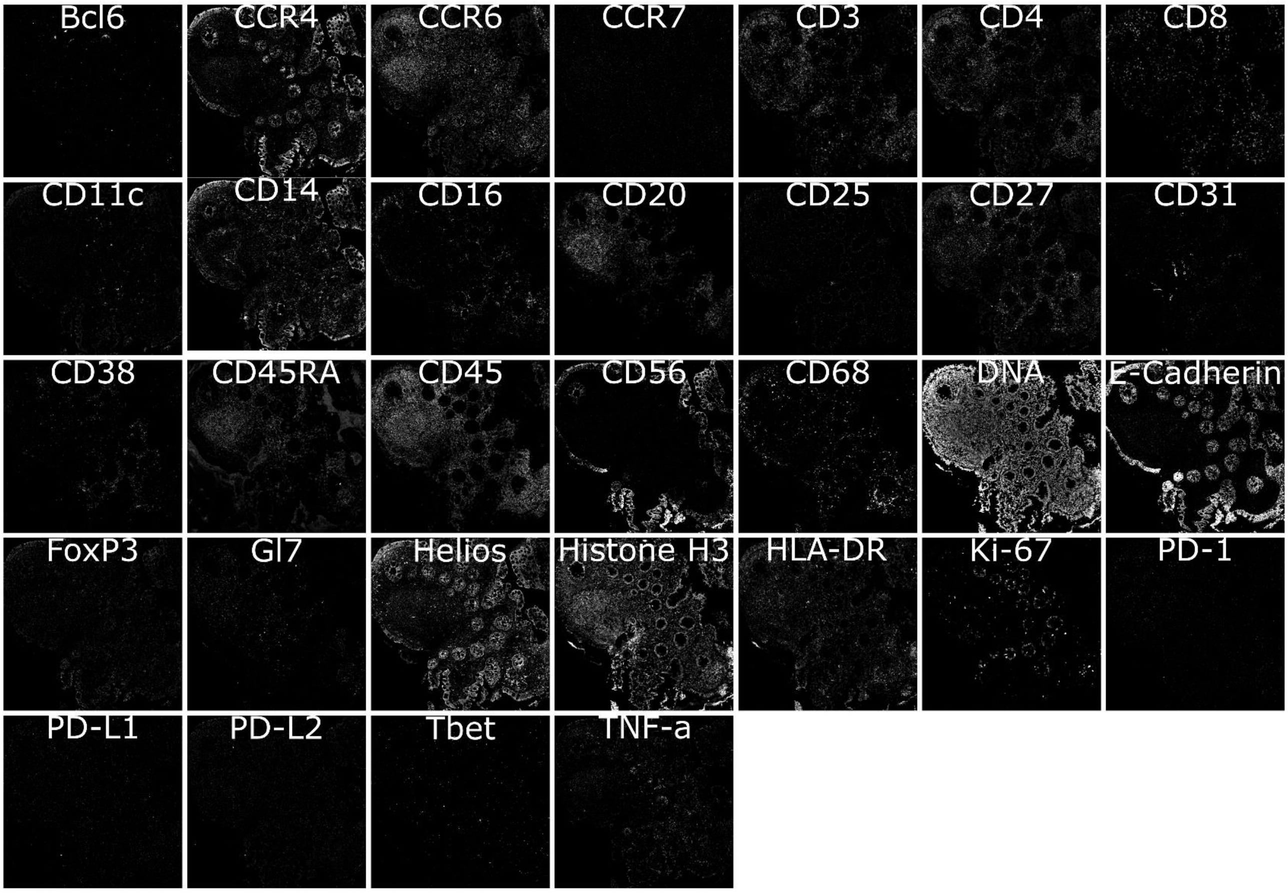

B

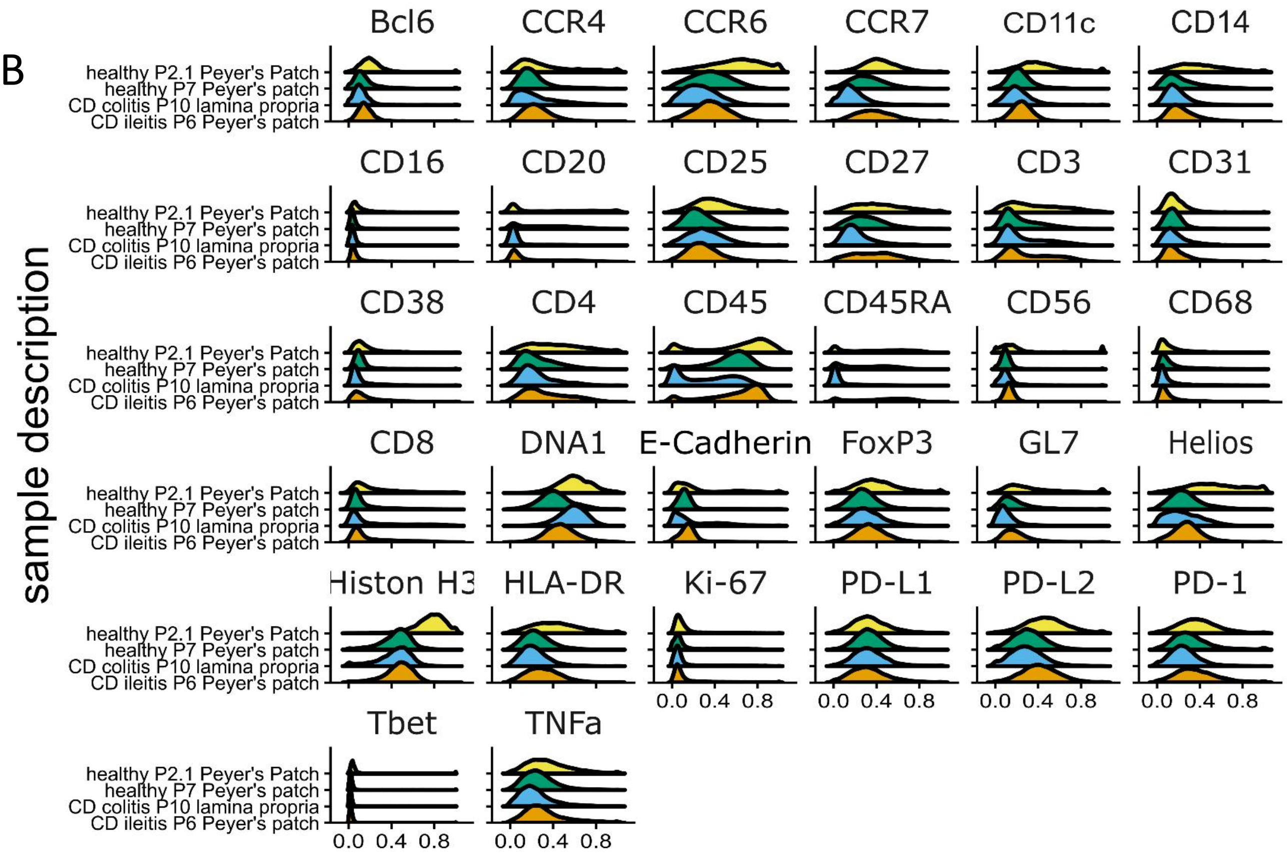

Supplemenatary Figure 1: Single marker expression values. (A) shows single marker staining for sample “healthy patient 2.1” Peyer’s patch. (B) shows intensity distributions for all markers after single cell segmentation for four randomly selected patients as indicated.

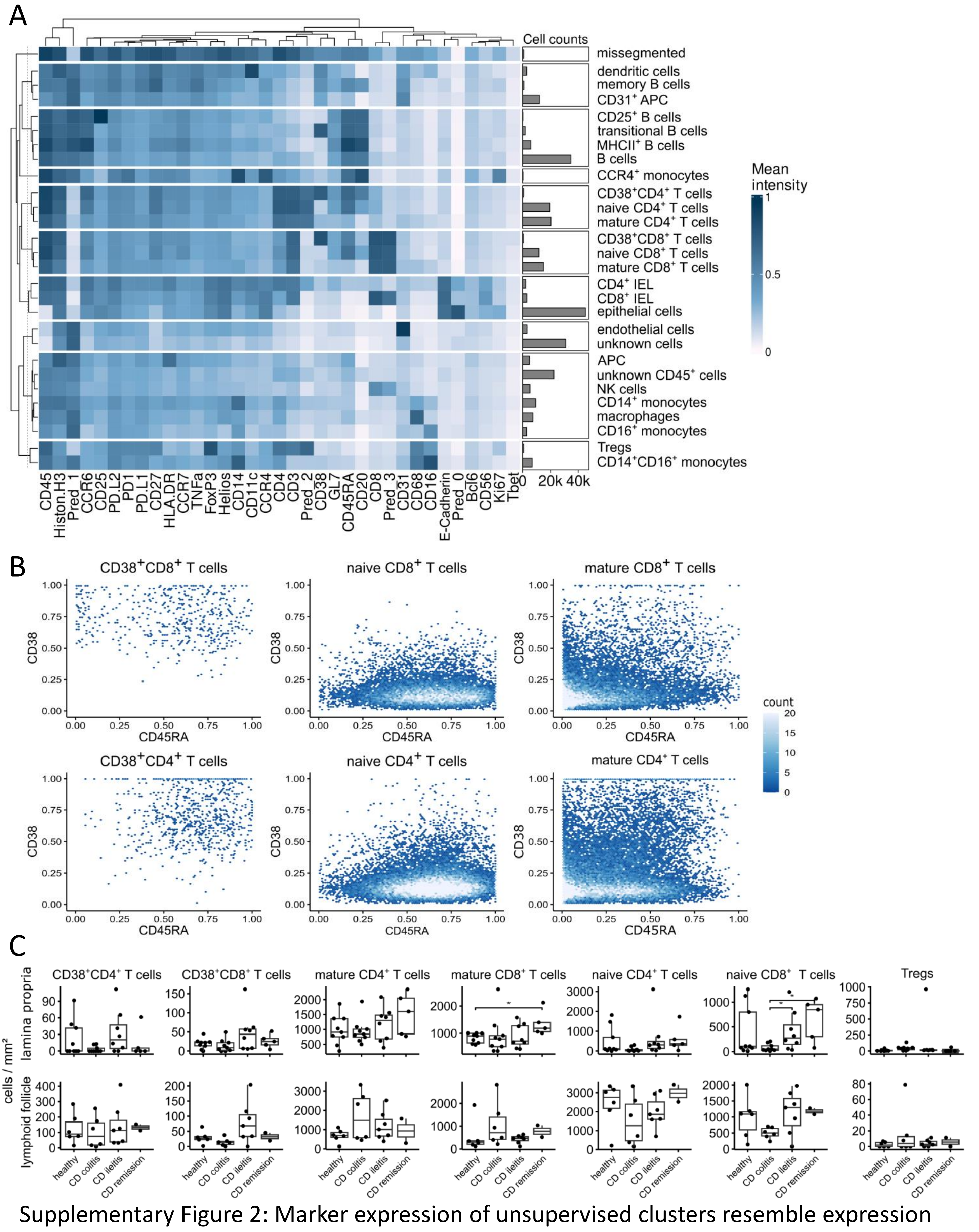

Supplementary Figure 2: Marker expression of unsupervised clusters resemble expression patterns of known cell populations. (A) Heatmap shows expression patterns for all annotated cell clusters. (B) Scatterplots showing expression of CD45RA and CD38 for identified major T cell subpopulations. (C) Cell density analysis of selected T cell populations (same populations as in Fig. 3C). For group-wise comparison, Wilcoxon test with Benjamini-Hochberg correction was used and only significant results are shown. *: $p \leq 0.05$

A

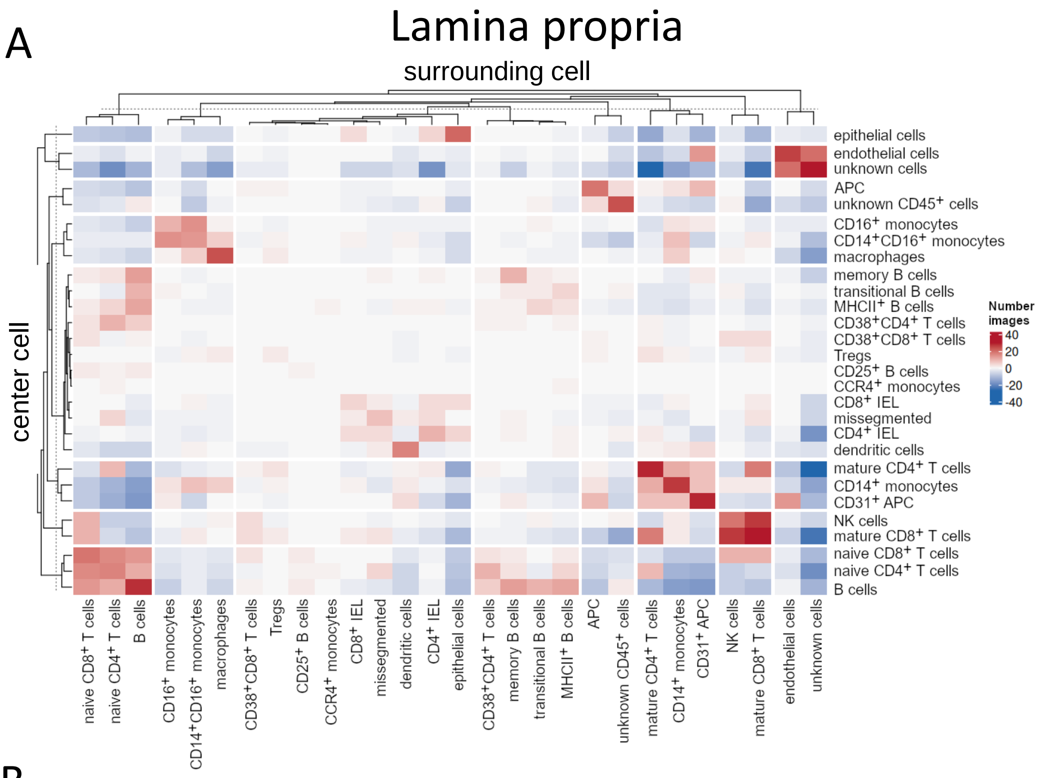

B

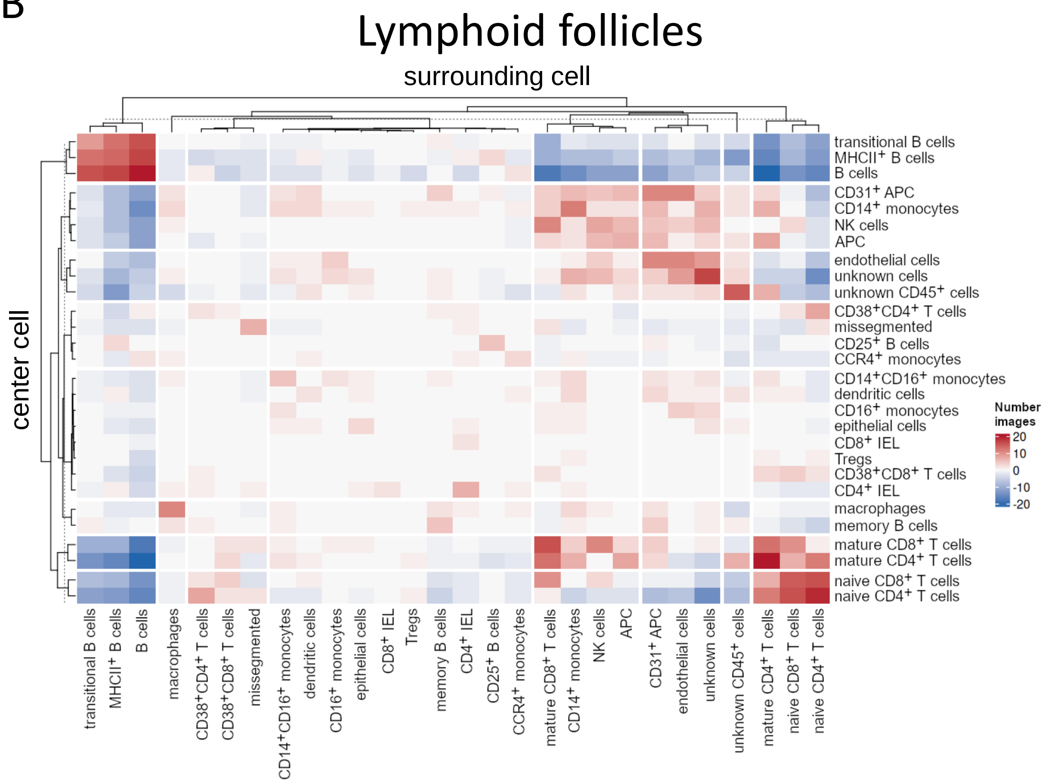

Supplementary Figure 3: Cell-Cell interactions differ significantly between lamina propria and lymphoid follicles. Heatmaps showing the cell-cell interactions calculated for the lamina propria (A) and lymphoid follicle (B) annotations separately including all cell populations.

A

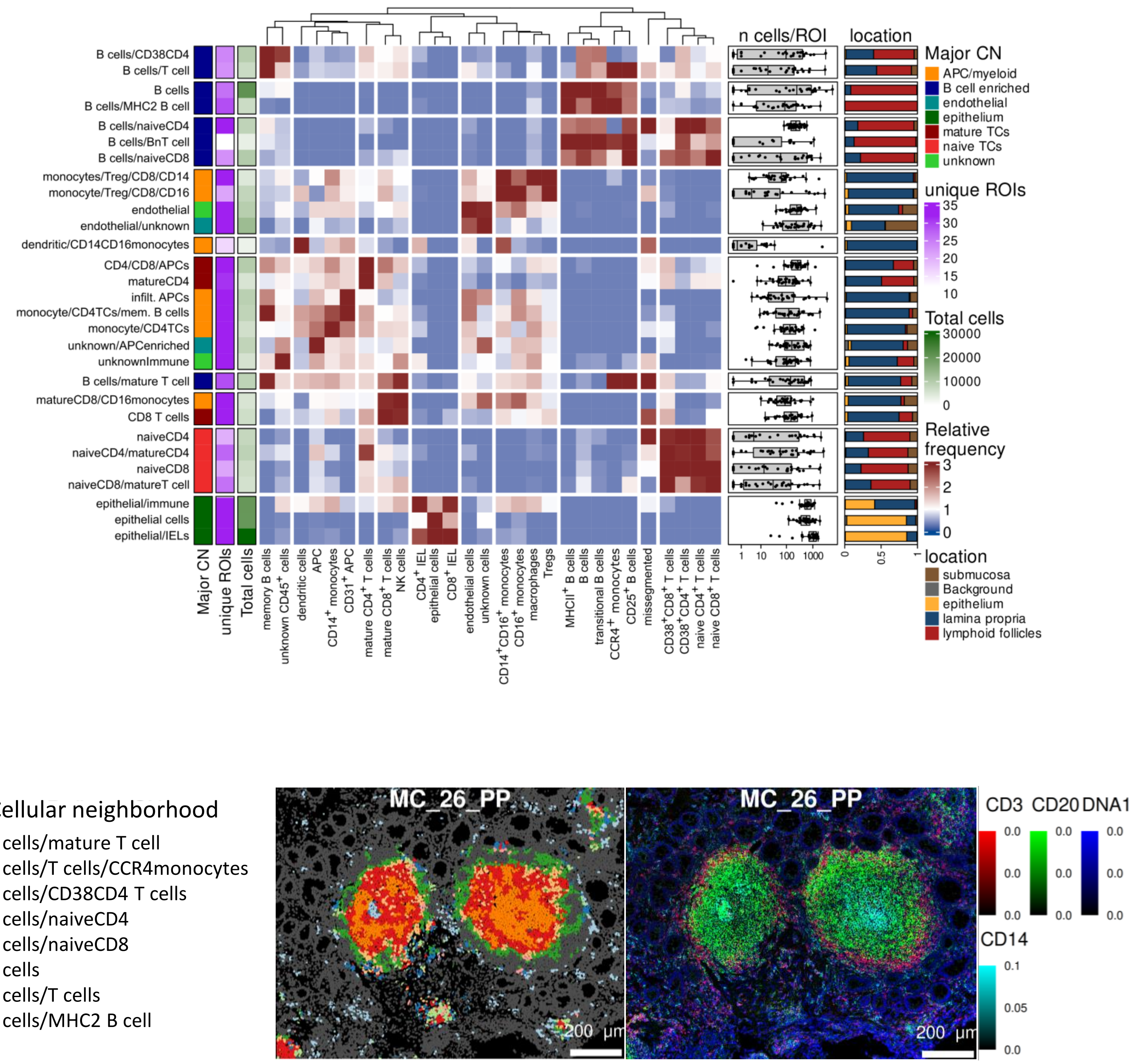

Supplementary Figure 4: Cellular neighborhoods representing cellular niches recapitulate tissue structures such as lymphoid follicles. (A) Cellular neighborhoods were identified by k-means clustering of the cell frequencies around each cell and clusters were subsequently annotated based on the enrichment of different cell populations as shown in the heatmap. (B) shows the distribution of different B cell enriched neighborhoods (left) and the expression of CD3 (red), CD20 (green), CD14 (cyan) and DNA (blue) (right panel).

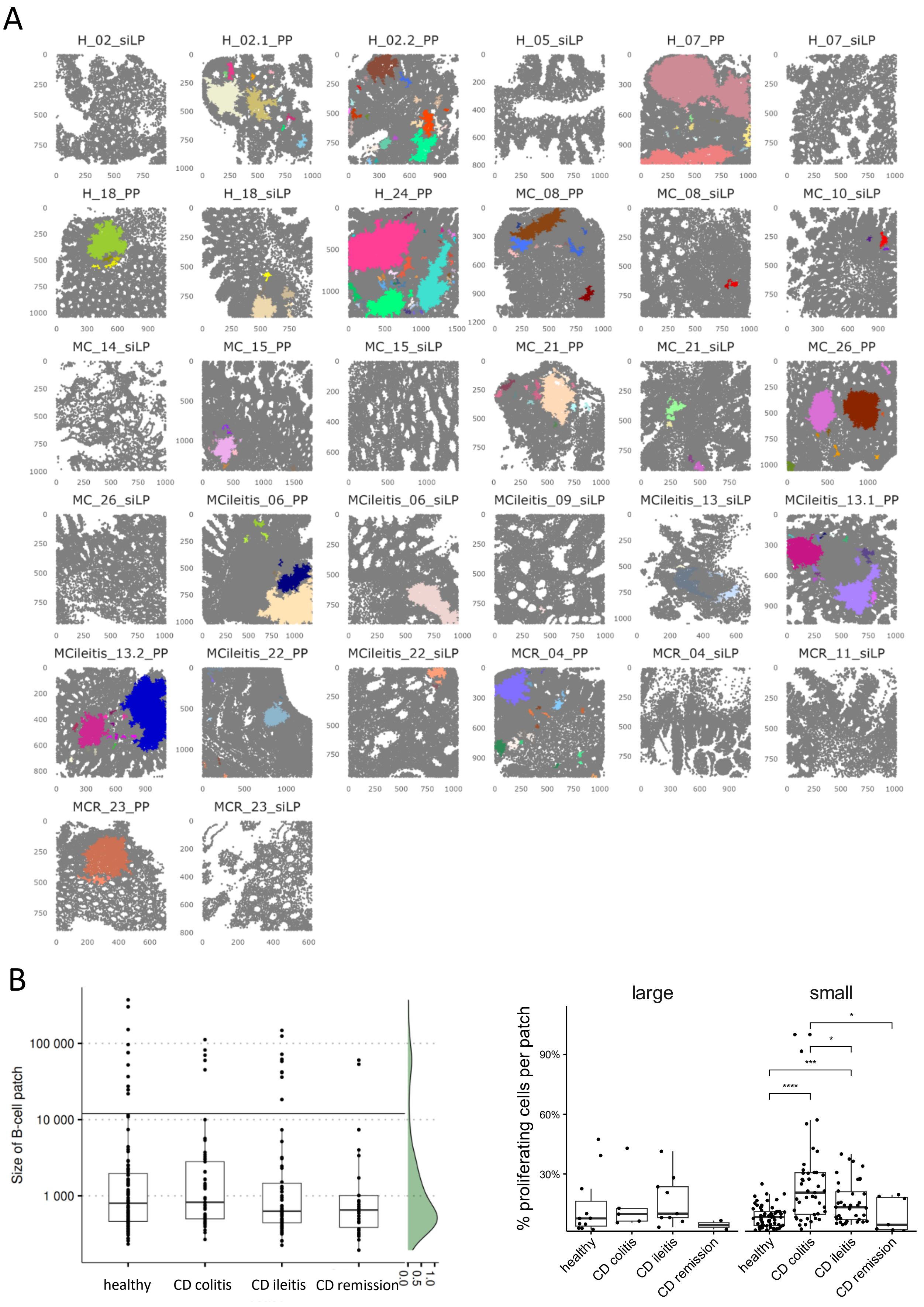

Supplementary Figure 5: B cell patch detection and size distribution. (A) shows all detected patches randomly color coded for all images . (B) shows the size distribution across different patient groups. (C) Percentage of proliferating cells ($Ki67^+$) was calculated for each detected patch. For group-wise comparison, Wilcoxon test with Benjamini-Hochberg correction was used and only significant results are shown. *: $p \leq 0.05$, **: $p \leq 0.01$, ***: $p \leq 0.001$, ****: $p \leq 0.0001$

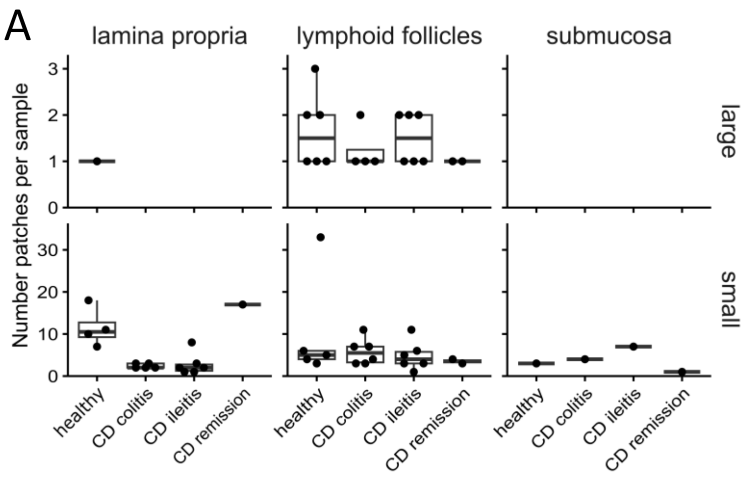

Supplementary Figure 6: Number of detected B cell patches per sample and annotated tissue area. (A) Cell number per patch and tissue segment was counted and patch was assigned to the tissue area with the highest cell count (to locate patches on borders i.e. between lamina propria and lymphoid follicles). Number of follicles was calculated per sample and separate for small and large follicles (see Fig. 5B, Suppl. Fig. 4B)).

Supplementary Table 1: Antibody list

Antibody	Metal	Clone	Supplier
CD38	141Pr	EPR4106	Standard BioTools
CD14	144Nd	EPR3653	
T-bet	145Nd	D6N8B	
CD16	146Nd	EPR16784	
Bcl-6	147Sm	K112-91	
IFNγ	148Sm	EPR21704	Abcam (Cambridge, UK)
CD194 (CCR4)	149Sm	L291H4	Standard BioTools
PD-L1	150Nd	E1L3N	
CD31	151Eu	EPR3094	
CD45	152Sm	D9M8I	
CD56	153Eu	CMSSB	Invitrogen (Thermo Fisher Scientific, Waltham, MA, USA)
CD11c	154Sm	Polyclonal	Standard BioTools
FoxP3	155Gd	236A/E7	
CD4	156Gd	EPR6855	
E-Cadherin	158Gd	24E10	
CD68	159Tb	KP1	
Helios	160Gd	MAB73091	R & D System (Minneapolis, MN, USA)
CD20	161Dy	H1	Standard BioTools
CD8a	162Dy	D8A8Y	
CD196 (CCR6)	163Dy	Polyclonal	
PD-1	165Ho	EPR4877(2)	
CD45RA	166Er	HI100	
CCR7	167Er	3D12	Invitrogen
Ki-67	168Er	B56	Standard BioTools
TNFα	169Tm	M1-C4	MilliporeSigma (Merck, St. Louis, MO, USA
CD3	170Er	Polyclonal, C-Terminal	Standard BioTools
CD27	171Yb	EPR8569	
PD-L2	172Yb	D7U8C	
GL7	173Yb	GL7	Invitrogen
HLA-DR	174Yb	YE2/36 HLK	Standard BioTools
CD25	175Lu	EPR6452	
Histone 3	176Yb	D1H2	
Iridium	191/193Ir	-	Standard BioTools

Supplementary Table 2: Enrichment of different cell subsets for each cellular neighborhood. Most prominent and dominant cell type was used for final label of neighborhood.

cellular neighborhood	(selected) enriched cell types
epithelial/immune	epithelial cell, several immune cells
epithelial cells	epithelial cells
epithelial/IELs	epithelial cells, $CD4^+$ IELs, CD8 IELs
monocytes/Treg/CD8/CD14	macrophages, Tregs, $CD14^+$ monocytes, mature $CD8^+$ T cells
monocyte/Treg/CD8/CD16	Tregs, $CD16^+$ monocytes, $CD14^+CD16^+$ monocytes, mature $CD8^+$ T cells
infilt. APCs	$CD31^+$ APC
monocyte/CD4TCs/mem. B cells	$CD31^+$ APC, memory B cells, mature $CD4^+$ T cells, monocytes
monocyte/CD4TCs	CD14+ monocytes, mature CD4+ T cells, APC, macrophages
unknown/APCenriched	unknown cells, APC
unknownImmune	unknown $CD45^+$ cells
endothelial	endothelial cells, unknown cells, some monocyte subsets
endothelial/unknown	endothelial cells, unknown cells
dendritic/CD14CD16monocytes	dendritic cells, $CD14^+CD16^+$ monocytes
matureCD8/CD16monocytes	mature $CD8^+$ T cells, NK cells, $CD16^+$ monocytes, unknown $CD45^+$ cells
CD8 T cells	mature $CD8^+$ T cells, NK cells
B cells/CD38CD4	memory B cells, unknown $CD45^+$ cells, $CD38^+CD4^+$ T cells, B cells, transitional B cells
CD4/CD8/APCs	mature $CD4^+$ T cells, mature $CD8^+$ T cells, $CD14^+$ monocytes, APC, $CD31^+$ APC
matureCD4	mature $CD4^+$ T cells
naiveCD4	naive $CD4^+$ T cells, $CD38^+CD4^+$ T cells, $CD38^+CD8^+$ T cells
naiveCD4/matureCD4	naive $CD4^+$ T cells, mature $CD4^+$ T cells, $CD38^+CD4^+$ T cells, $CD38^+CD8^+$ T cells
naiveCD8	naive $CD4^+$ T cells, naive $CD8^+$ T cells, $CD38^+CD4^+$ T cells, $CD38^+CD8^+$ T cells
naiveCD8/matureT cell	naive CD4+ T cells, naive CD8+ T cells, CD38+CD8+ T cells, mature CD4+ T cells, mature CD8+ T cells
B cells/mature T cell	naive CD4 T cells, naive CD8+ T cells, CD38+ CD8+ T cells
B cells/T cell	memory B cells, B cells, $CD25^+$ B cells, $CCR4^+$ monocytes, $CD38^+CD4^+$ T cells
B cells	$MHCII^+$ B cells, B cells, transitional B cells
B cells/MHC2 B cell	$MHCII^+$ B cells transitional B cells, $CCR4^+$ monocytes
B cells/naiveCD4	naive $CD4^+$ T cells, $CD38^+CD4^+$ T cells, several B cell subsets
B cells/BnT cell	$MHCII^+$ B cells, B cells, transitional B cells, $CCR4^+$ monocytes, $CD38^+CD4^+$ T cells, naive $CD8^+$ T cells
B cells/naiveCD8	naive $CD8^+$ T cells, $CD25^+$ B cells, B cells
